# Improved Self-Supporting and Ceramifiable Properties of Ceramifiable EPDM Composites by Adding Aramid Fiber

**DOI:** 10.3390/polym12071523

**Published:** 2020-07-09

**Authors:** Dong Zhao, Wei Liu, Yucai Shen, Guodong Jiang, Tingwei Wang

**Affiliations:** 1 College of Material Science and Engineering, Nanjing Tech University, Nanjing 211816, China; 8113557343@njtech.edu.cn (D.Z.); 201861103043@njtech.edu.cn (W.L.); gdjiang@njtech.edu.cn (G.J.); 2 Jiangsu Collaborative Innovation Center for Advanced Inorganic Function Composites, Nanjing 211816, China; 3 Suqian Advanced Materials Institute of Nanjing Tech University, Suqian 223800, China

**Keywords:** EPDM, ceramifiable, aramid fiber, self-supporting, thermal stability

## Abstract

Ceramifiable ethylene propylene diene monomer (EPDM) composites with fiber network structures were prepared by using aramid fiber (AF), ammonium polyphosphate (APP), and silicate glass frits (SGF). The effect of AF on the curing characteristic of the ceramifiable EPDM composites was studied. The morphology of AF in the composites system was observed by optical microscopy (OM) and scanning electron microscope (SEM). The effects of the observed AF network structures on the solvent resistance, mechanical properties, ablative resistance, self-supporting property, and ceramifiable properties of the composites were investigated. Results suggested that the existence of the AF network structure improved the vulcanization properties, solvent resistance, thermal stability, and ablative resistance of the EPDM composites. An excellent self-supporting property of the EPDM composites was obtained by combining the formation of the AF network and the formation of crystalline phases at higher temperature (above 600 °C). The thermal shrinkage performance of AF and the increased thermal stability of the EPDM composites improved the ceramifiable properties of the EPDM composites.

## 1. Introduction

Polymeric ablative materials are usually used for insulation and fire prevention in electric power and propulsion systems [1,2,3]. Ethylene-propylene-diene monomer (EPDM) represents one of the best matrices for polymeric ablative materials [4,5,6]; the thermal stability and electrical insulation of EPDM is comparable to or higher than that of other rubbers [7]. Therefore, EPDM as a synthetic rubber is used in construction, electrical insulation, automobile manufacturing, and even propulsion systems of solid rocket motors [8,9,10,11]. However, as a type of thermal protection material, the dripping and bending behaviors of EPDM composites have been the most serious drawback, which can cause the composite to lose its thermal protection function and even ignite other flammable materials. Thus, it is important to develop a novel EPDM composite with self-supporting and anti-dripping properties.

In addition, EPDM composite has been often used in the thermal protection field because of its high char yield, which increases the ablation resistance and the thermal insulation performance [3,8,10,12]. With the development of aerospace solid propulsion technology, the thermal protection material will also be mechanically eroded by the action of condensed high speed solid alumina particles produced during the combustion of modern aluminized solid rocket motor propellants [13,14]. Thus, this requires a thermal protection material with higher anti-erosion and ablation resistance properties.

Ceramifiable polymer composites possess the performance features of polymers at room temperature and of ceramics at high temperatures [15,16,17,18,19]. Bennett and Young [20] reported that for the glass ceramic containing boron, in which B_2_O_3_ can be formed from borates and boric acid at high temperatures, a glassy protective layer emerged. Novel laponite-armored hollow composite particles (LHCPs) with a pre-organized “house-of-cards” structure were synthesized by Zhang [21]. It was revealed that the ceramifiable silicone rubber with laponite platelets as fillers can maintain its shape and mechanical strength in the range of 500–900 °C. The ceramic residues of ceramifiable silicone rubber can also act as a heat transport barrier that protects the underlying material from incoming heat flux [22]. In addition, the fire resistance and ablation resistance of the ceramifiable polymer composites at high temperatures could be further improved by forming crystalline phases. In our previous work [23,24], a novel ceramifiable ethylene-vinyl acetate (EVA) composite and a novel ceramifiable silicon rubber composite were successfully prepared. A novel ceramization mechanism of liquid-solid transition was also proposed. However, the formation of inorganic crystalline phases tends to happen at high temperatures (over 600 °C). Before the formation of crystalline phases, polymer composites are often too weak to keep their original shape. A cross-linking modification was often considered to enhance the thermal stability of the composites [25,26], and ceramifiable EVA composites with improved self-supporting and ceramifiable properties were obtained using a cross-linking strategy [27]. However, there are still dripping and bending behaviors during the ablation process for cross-linked ceramifiable EPDM composites.

Aramid fibers (AF) were often used as a low-density reinforcement due to their high thermal capacity, high chemical stability, high fire resistance, and high ablation resistance [28,29,30,31]. Qin et al. [32] prepared a type of ceramifiable EPDM rubber composites with EPDM, AF, flux, and other fillers and focused on the investigation of the effect of different fluxes on the formation of ceramic structure. Chen et al. [33] found that AF could improve the ablative resistance of EPDM vulcanizate effectively compared to basalt fiber and carbon fiber. Maurizio et al. [5,34] prepared a type of EPDM thermal protective material for a solid rocket motor. They found that AF had a better interfacial compatibility with the EPDM matrix than silicon fiber. In addition, AF formed a carbon framework at high temperatures, which improved the ablative resistance of the composite. Li et al. [35] studied the ablation and erosion characteristics of EPDM composites under realistic solid rocket motor operating conditions. They found that the combined use of silica and AF in EPDM composites can improve the heat-shielding performance of the char layer. In this work, in order to significantly improve the thermal stability of the ceramifiable EPDM composite before the formation of crystalline phases at high temperature, a novel ceramifiable EPDM composite was prepared by adding AF, ammonium polyphosphate (APP), and silicate glass frits (SGF) as a ceramic precursor. We aimed to improve the self-supporting performance and optimize the ceramifiable properties of EPDM composite by forming an AF network structure.

## 2. Experimental

### 2.1. Materials

Ethylene propylene diene monomer (EPDM) (DuPont, Nordel IP-4725P) and aramid fiber (AF) (ALKEX, AF-1000) were supplied by Shanghai Academy of Aerospace Propulsion Technology. Silicate glass frits (SGF) (500 mesh) was provided by Donggu New Material Co., Ltd. (Foshan, China). Ammonium polyphosphate (APP) (crystalline form II, polymerization degree exceeds 1500) was supplied by Zhengzhou Hao Rong Chemical Products Co., Ltd. (Zhengzhou, China). Sulfur (S) was purchased from Merck, Germany. Dicumyl peroxide (DCP) was supplied by He Fei An Bang Chemical Co., Ltd. (Hefei, China).

### 2.2. Sample Preparation

EPDM and other materials were compounded on a two-roll mill, as listed in Table 1. The rolls were set to a temperature of 50 °C. EPDM was first mixed with AF, and then the SGF and APP were added. Finally, the remaining additives (DCP and S) were added. The total mixing time was 15 min. The vulcanization parameters, curing time (*t*_90_), and maximum (*M*_H_) and minimum (*M*_L_) torque were determined using the Dynamic Moving Die Rheometer (MDR 2000, Wuxi Liyuan Electronic & Chemical Equipment Co., Ltd., Wuxi, China). For characterization of the ceramifiable EPDM composites, sheets with thicknesses of 2 and 10 mm were cured in a compression mold at a temperature of 180 °C with a pressure of 10 MPa for 15 min.

### 2.3. Characterization

The microstructure of the cured ceramifiable EPDM composites and the distribution of the AF in the system were observed using a metallographic microscope (Axio Observer A1m, Carl Zeiss Jena Company, Jena, Germany).

Samples with the dimensions of 20 mm × 20 mm × 2 mm were immersed in 40 mL of tetrachloromethane solution at 25 °C for 12 h. The liquid absorbency (A) was calculated as follows with Equation (1):

*A* = (*m*_t_ − *m*_0_)/*m*_0_ × 100% (1)

where *A* is the liquid absorbency, and *m*_0_ and *m*_t_ are the weight of the dry sample and swollen sample, respectively. In addition, the dimension of the sample before and after swelling was also recorded.

Tear tests were carried out using a universal testing machine (CMT 5254, Shenzhen SANS Testing Machine Co., Ltd., Shenzhen, China), under a stable rate of 100 mm/min, according to the GB/T529-2008 standard. The test results were the average of at least five specimens.

All samples (125 mm × 13 mm × 2 mm) were measured using the vertical burning test instrument (CZF-3) (Nanjing Jiangning analytical instrument factory, Nanjing, China) according to the GB/T10707-2008 standard.

The self-supporting property of the ceramifiable EPDM composites was tested by measuring the bending angles of the composites after firing at different temperatures for 30 min in a muffle furnace. The test method is as follows: (1) firstly, the sample (50 mm × 5 mm × 2 mm) was placed on a refractory brick. Moreover, the long axis of the sample was perpendicular to the edge of the brick, and 20% of the length of the sample extended from the edge of the brick; (2) then, all samples were fired at 600, 700, 800, and 900 °C; (3) finally, the bending angle of the residue to its original position was obtained [24].

The linear ablation rate test under a oxyacetylene torch was conducted following the GJB323A-96 standard. All specimens were vertically subjected to the flame gun with a heat flux of 4110.0 KW/m^2^. Gas pressures of O_2_ and C_2_H_2_ were 0.4 and 0.095 MPa, respectively, and the gas fluxes were 1512 and 1116 L/h, respectively. The oxyacetylene gun tip was 2 mm in diameter, and its distance from the sample was 10 cm. Linear ablation rates of the specimens were obtained according to Equation (2):

*A* = (*d*_t_ − *d*_0_)/*t* (2)

where *A* is the linear ablation rate; *d*_t_ and *d*_0_ are the thickness of the specimen at the center region before and after ablation, respectively; and *t* is the ablation time. Three specimens were tested for each of the samples.

The flexural strength of residues was tested on a universal testing machine (UH 6502, Youhong Measurement and Control Technology Co., Ltd., Shanghai, China) by the three-point bend method at a stable rate of 0.5 mm/min, according to the GB/T9596-2006 standard.

Linear shrinkage was obtained according to the length of the sample before and after firing at high temperatures. The linear shrinkage was calculated using Equation (3):

*L* = (*L*_0_ − *L*_d_)/*L*_0_ × 100% (3)

where *L* is linear shrinkage (%); and *L*_0_ and *L*_d_ are the length of the specimen before and after firing, respectively.

Thermogravimetric analysis (TGA) was performed on a TA Instruments (STA449C) at a heating rate of 10 °C/min under N_2_ atmosphere. All composites were heated in a temperature range from room temperature to 800 °C. Moreover, pure AF was heated from room temperature to 700 °C.

The surface morphology of the residue and the state of AF in the residue were observed on a scanning electron microscope (SEM) (JSM-6510, Jeol, Tokyo, Japan) with an acceleration voltage of 15 kV.

The phase composition of the residue powders were characterized through an X-ray diffraction instrument (Rigaku Corporation, Akishima, Tokyo, Japan) with Cu Kα radiation in the 2θ range of 10°–80° at a scan rate of 10°/min.

The fourier transform infrared spectroscopy (FTIR) spectra of residue powders were recorded with a Nicolet spectrometer (NEXUS670, Thermo Fisher, Waltham, MA, USA) with a range of 4000–400 cm^−1^.

## 3. Results and Discussion

### 3.1. Curing Properties of Ceramifiable EPDM Composites

**Figure 1:**
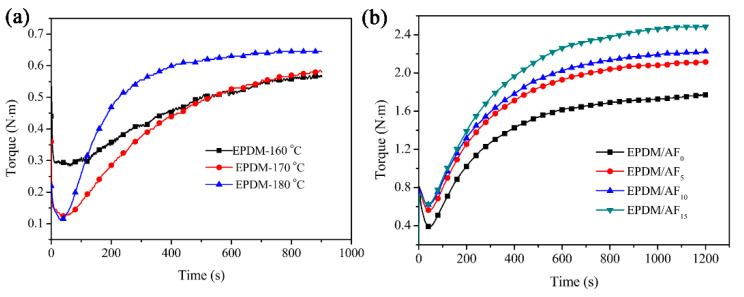
Curing curves of the pure EPDM (**a**) and ceramifiable EPDM composite (**b**).

**Figure 2:**
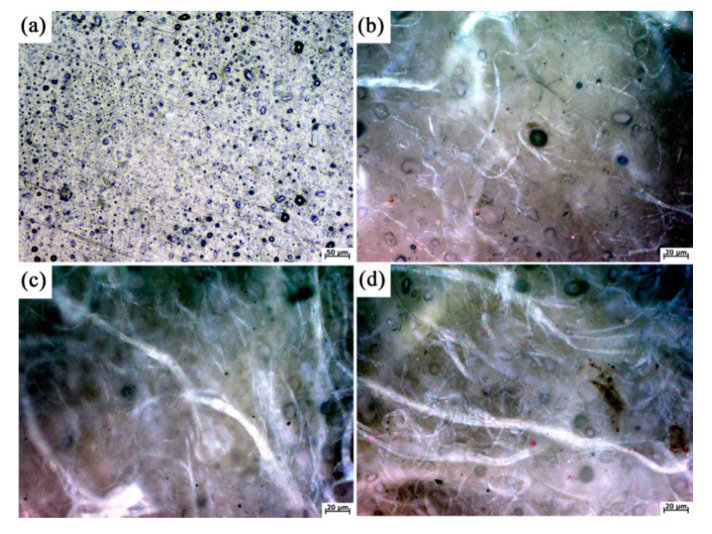
Optical microscopy (OM) photographs of EPDM/AF_0_ (**a**), EPDM/AF_5_ (**b**), EPDM/AF_10_ (**c**), and EPDM/AF_15_ (**d**).

The vulcanization properties of pure EPMD at different temperatures are shown in Figure 1a and Table 2. It was observed that ceramifiable EPDM has a different optimum cure time at different temperatures. Under the curing time of 900 s, EPDM was not in the plateau of vulcanizing curves at 160 and 170 °C, while the state of vulcanized EPDM was in the plateau of vulcanizing curves at the temperature of 180 °C. Therefore, the curing condition of 180 °C and 900 s was applied for ceramifiable EPDM composites. Figure 2b and Table 3 show the curing properties of the ceramifiable EPDM composites. The addition of inorganic fillers (SGF and APP) and AF into the EPDM rubber increased its minimum torque (*M*_L_) and maximum torque (*M*_H_), which can be ascribed to the inorganic reinforcing fillers [36,37]. Table 3 shows that the *M*_H_ value gradually increased with further addition of AF, showing again the reinforcing effect of AF. The value of Δ*M* (*M*_H_ − *M*_L_) increased as the content of AF increased, indicating that reduced slippage between the polymer chains may occur [38].

### 3.2. Surface Morphology of Ceramifiable EPDM Composites

In order to observe the effect of AF on the ceramifiable EPDM composites, the morphology of the ceramifiable EPDM composites for various contents of AF were characterized using optical microscopy (OM) (Figure 2). The OM micrographs of the surface of EPDM/AF_10_ and EPDM/AF_15_ indicate that the AF was evenly distributed. There were many superfine fibers sticking on the main trunk fiber, which could promote the formation of a fiber network in ceramifiable EPDM composites.

### 3.3. Swelling Property of Ceramifiable EPDM Composites

**Figure 3:**
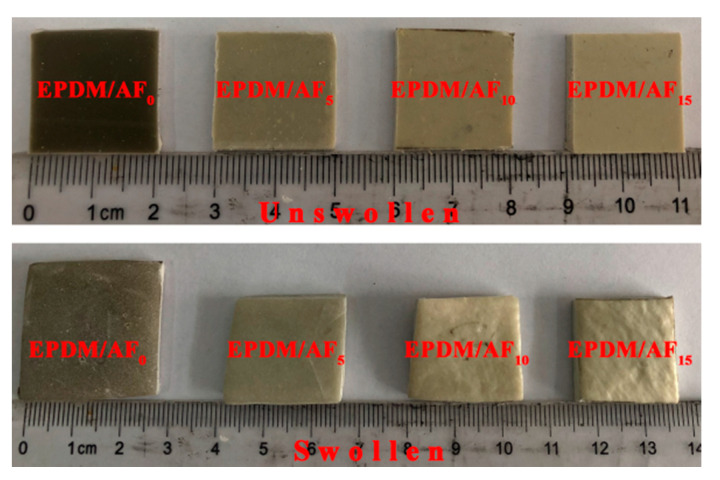
Digital photos of ceramifiable EPDM composite before and after swelling in pure tetrachloromethane.

The swelling test results of the ceramifiable EPDM composites are presented in Figure 3 and Table 4. It is clear that the sample of EPDM/AF_0_ showed the most serious dimensional changes and the highest liquid absorbency among all the composites. In tetrachloromethane solvent, the percentage of liquid absorbency significantly decreases with increasing AF content from 5–15 wt %. It can be observed that there was little change in the dimension of sample EPDM/AF_15_, as shown in Figure 3. It is widely accepted that the swelling is directly related to the cross-linking density of molecular chains [39,40,41], and less solvent uptake or penetration into the blends indicated higher cross-linking density. However, there is no cross-linking reaction between EPDM molecular chains and AF fiber; the lower solvent uptake or penetration into the blends could be attributed to the AF fiber, which restricted the slippage between the polymer chains [41]. Therefore, the solvent cannot easily penetrate into the blends. Swelling test results showed that the addition of AF enhanced the interaction within the system of the composite. This enhanced interaction within the system of the material might be able to improve the self-supporting and anti-dripping properties at high temperatures.

### 3.4. Mechanical Properties of Ceramifiable EPDM Composites

Table 5 shows that there was an obvious increase in tear strength from 14.85 N/m to 22.02 N/m for ceramifiable EPDM composites when the content of AF increases from 5 to 15 wt %. The improvement of tear strength was due to the addition of AF for absorbing energy and blocking crack-growth during the tearing process [42].

### 3.5. Anti-Dripping and Self-Supporting Properties of Ceramifiable EPDM Composites

It can be seen from Table 6 that samples EPDM/AF_0_ and EPDM/AF_5_ had serious melt dripping, while EPDM/AF_10_ and EPDM/AF_15_ did not show any melt dripping. However, no samples achieved any classification in the UL 94 test, as they did not extinguish within a short time period of the defined specimen after removing the Bunsen-burner-type ignition source.

To characterize the self-supporting property, the bending angles of ceramifiable EPDM composites firing at high temperatures were tested. As shown in Table 7, when the firing temperature increased from 700 to 900 °C, the bending angles of the residues obtained by firing the same sample increased in different degrees. Under the same firing condition, the bending angles of the sample decreased with the increasing content of AF. The occurrence of bending behavior was ascribed to soften and molten of the materials at high temperatures. Bending deformation of the ceramifiable composites can be suppressed by forming a crystalline phase at high temperatures (above 600 °C) [24,27]. Before the formation of the crystalline phase, the polymer composite is often too weak to support its own weight. The addition of AF could significantly enhance the thermal stability of the EPDM composites by forming a fiber network structure, leading to a remarkable improvement of the self-supporting property at high temperatures.

### 3.6. Linear Ablation Property of Ceramifiable EPDM Composites

Table 8 shows the ablation rates of the ceramifiable EPDM composites after exposure to the oxyacetylene torch flame for 20 s under 4110.0 KW/m^2^ of heat flux. Sample EPDM/AF_0_ was burned through, as shown in Figure S3 (Supplementary Materials). However, the linear ablation rate of composites decreased from 0.28 mm/s to 0.23 mm/s when the AF content in the samples increased from 5 to 15 wt %, and their back sides maintained the original features. This indicated that the introduction of AF greatly improved the ablation performance [42].

### 3.7. Flexural Strength and Linear Shrinkage of Ceramifiable EPDM Composites

**Figure 4:**
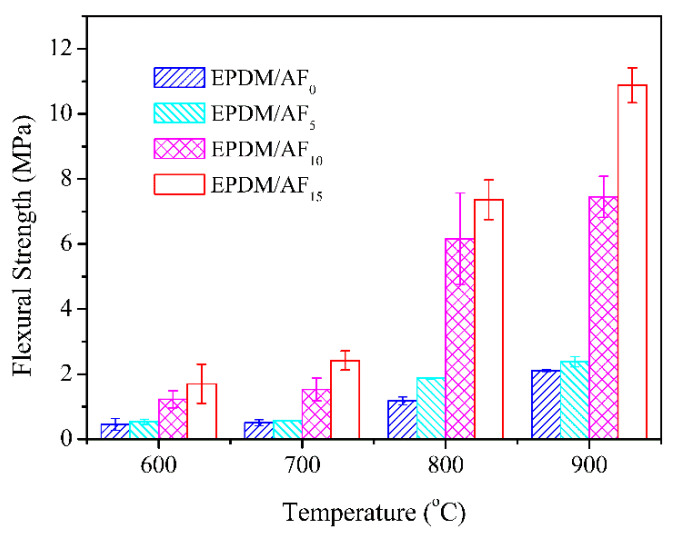
Flexural strength of ceramifiable EPDM composites at different temperatures.

**Figure 5:**
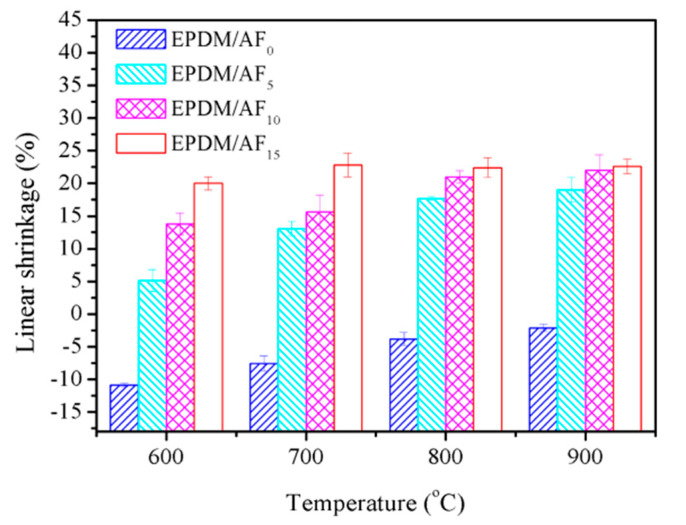
Linear shrinkage of ceramifiable EPDM composites at different temperatures.

Figure 4 and Figure 5 present the effects of AF on the flexural strength and linear shrinkage of the ceramifiable EPDM composites fired at different temperatures. In the temperature range of 700–900 °C, the flexural strength of all samples increased as the firing temperature increased. This phenomenon was in accordance with the ceramifiable EVA composites [43]. It was noteworthy that the flexural strength improved as the AF content increased in EPDM/AF_5_, EPDM/AF_10_, and EPDM/AF_15_ under the same pyrolysis conditions. At 900 °C, the flexural strength of the ceramic residue reached the maximum value of 10.88 MPa, exhibiting excellent mechanical properties.

EPDM/AF_0_ exhibited linear expansion at various temperatures owing to the fast decomposition of the EPDM matrix and APP, which resulted in poor flexural strength. In the case of EPDM/AF_5_, EPDM/_10_, and EPDM/_15_, the linear shrinkage was positive at various temperatures, indicating that a degree of sintering occurred in these samples. The linear shrinkage of the sample under the same firing conditions increased with increasing AF. In addition, taking samples EPDM/AF_0_ and EPDM/AF_10_ as examples, their surface morphology fired at 600, 700, 800, and 900 °C is shown in Figure S1 (Supplementary Materials). These results indicated that the addition of AF can improve the compactness of the residue. These results correspond with the flexural strength results.

### 3.8. Thermogravimetric Analysis

**Figure 6:**
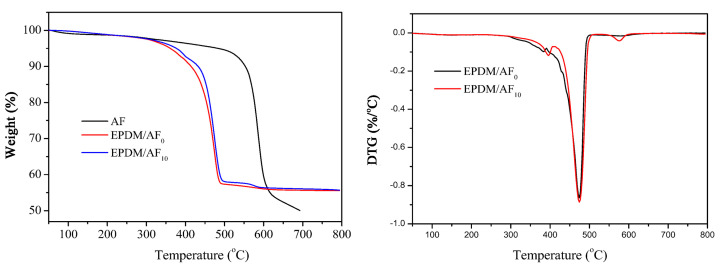
Thermogravimetry (TG) and differential thermogravimetry (DTG) curves of samples AF, EPDM/AF_0_ and EPDM/AF_10_ under N_2_.

TGA data of the samples under N_2_ atmosphere are presented in Figure 6 and Table 9. A characteristic temperature was used to evaluate the thermal stability: *T*_5%_ (temperature when weight loss reaches 5 wt %). For pure AF, it can be seen that the *T*_5%_ is 481.23 °C, and the residual mass at 500 °C was 94.49 wt %. It was possible to contribute to an excellent self-supporting performance of EPDM/AF composites at high temperatures. In addition, it could be seen that the thermal degradation of EPDM/AF_0_ and EPDM/AF_10_ both involved two main steps. There was a slight difference in the first steps of degradation between EPDM/AF_0_ and EPDM/AF_10_. EPDM/AF_10_ exhibited a higher *T*_5%_ degradation temperature (*T*_5%_ 378.39 °C), while EPDM/AF_0_ displayed a lower *T*_5%_ degradation temperature (T_5%_ 360.73 °C). For EPDM/AF_0_, the temperature at the maximum weight loss rate (*T*_max1_) in the main step was 381.57 °C, which was lower than the corresponding value of EPDM/AF_10_ (*T*_max1_ 394.16 °C). Compared with EPDM/AF_0_, the char residue of EPDM/AF_10_ increased from 56.74% to 57.62% at 500 °C. The occurrence of these behaviors could be attributed to the existence of the AF network structure, resulting in a higher thermal stability of the EPDM/AF system [44]. In addition, a weak thermal degradation step appeared between 550–600 °C owing to the slower pyrolysis of AF. This indicated that AF can still remain after the pyrolysis of EPDM and APP. Therefore, the addition of AF not only can improve the thermal stability of the sample before the formation of crystalline phases but also can increase the compactness of the residue.

### 3.9. SEM Analysis

**Figure 7:**
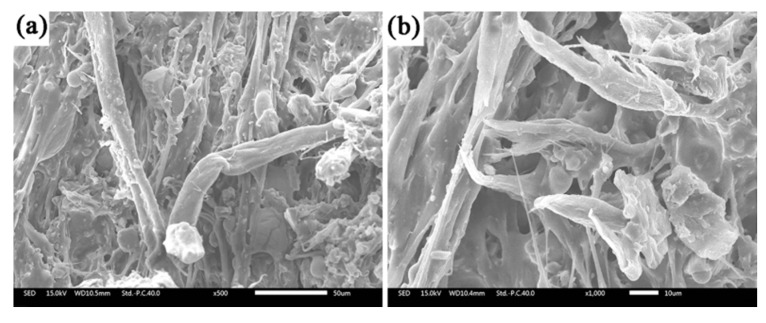
SEM of the sample EPDM/AF_10_ fired at 350 °C for 20 min: (**a**) ×500; (**b**) ×1000.

Figure 7 shows the SEM images of the sample EPDM/AF_10_ fired at 350 °C for 20 min. It clearly shows the existent form of AF in the cross section of residue, and the structure of AF is relatively undamaged. In addition, residues from SGF, APP, and EPDM decomposition attached to the fiber. Thus, anti-dripping and self-supporting properties of ceramifiable EPDM composites were improved owing to the increase in interaction between residues at high temperatures.

**Figure 8:**
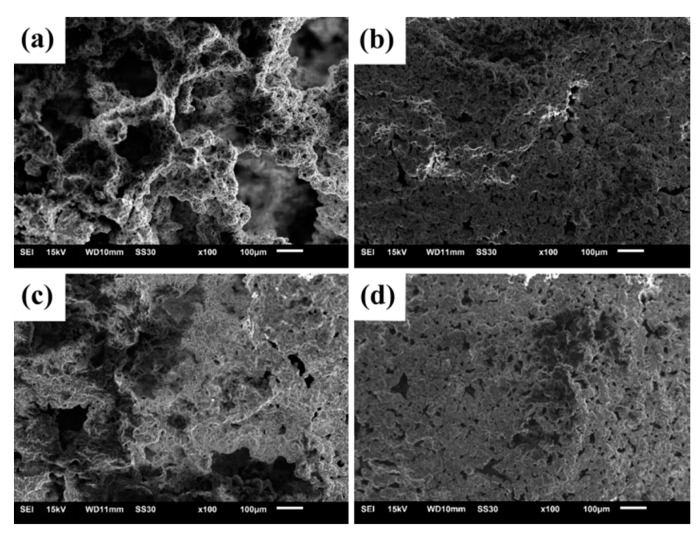
Surface images of samples: EPDM/AF_0_ after firing at 600 °C (**a**) and 800 °C (**c**); EPDM/AF_10_ after firing at 600 °C (**b**) and 800 °C (**d**).

The surface structures of the residues of EPDM/AF_0_ fired at 600 °C and 800 °C for 30 min are shown in Figure 8a,c. The surface of the residue fired at 600 °C exhibited many pores in Figure 8a. The formation of pores was attributed to the pyrolysis of APP and the EPDM matrix. However, when the sample fired at 800 °C, a more compact structure was formed, as observed in Figure 8c. Densification of the residue can be due to the strong liquidity of molten fillers at high temperatures. Therefore, the flexural strength increased with the increase in firing temperature, and this was also consistent with previous research on the EVA system [27].

It is noteworthy that the residues of EPDM/AF_10_ showed a more compact structure at corresponding temperatures compared with the residues of EPDM/AF_0_. As shown in Figure 8b,d, a dense microstructure was formed at 600 and 800 °C, which caused the improvement in the flexural strength of the fired samples. According to the results of EPDM/AF_0_ and EPDM/AF_10_, a possible mechanism for the formation of the compact surface structure of the EPDM/AF_10_ residue was suggested. For EPDM/AF_10_, the formation of the compact ceramic structure can be attributed to the existence of AF. During the processing of composites, the AF fibers were subjected to a degree of stretching and orientation. With the temperature increasing, AF will shrink slowly [5]. The shrinkage behavior of AF at high temperatures can be seen in Figure S2 (Supplementary Materials). Thermal shrinkage of AF will make the close-contacted fillers and EPDM matrix move together; thus, the addition of AF leads to the obvious shrinkage of ceramifiable EPDM composites at high temperatures and improves the sintering densification.

### 3.10. Mechanism for the Transformation to Ceramic at High Temperature

The existence of AF inhibited the dripping and bending of the ceramifiable EPDM composite at high temperature (500–600 °C). Theoretically, when the firing temperature is higher than the decomposition temperature of the AF, the material will tend to bend or melt drip due to the fusibility and flow properties of inorganic fillers [24,27]. On the contrary, the EPDM/AF system still showed a good self-supporting property over 600 °C.

**Figure 9:**
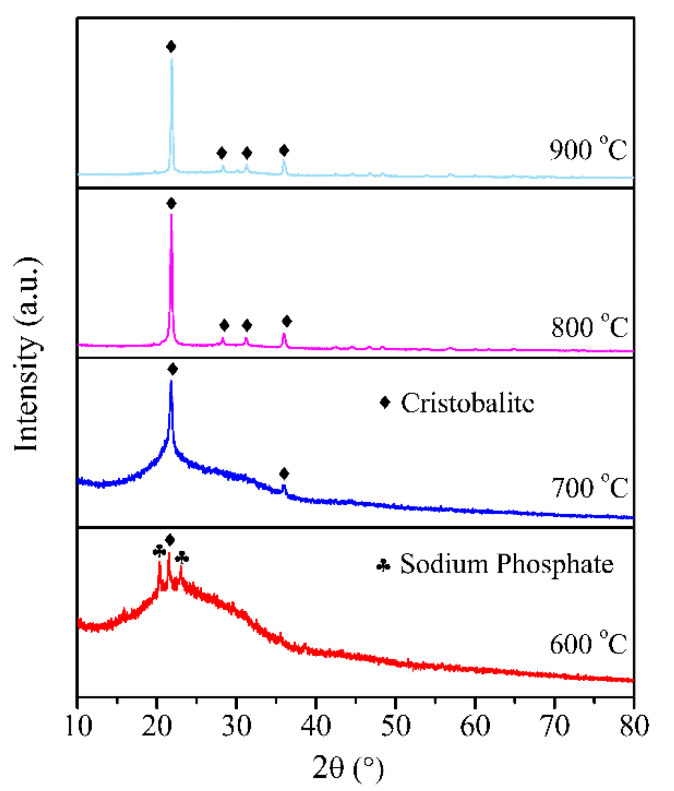
XRD patterns of residues of EPDM/AF_10_ fired at different temperatures.

**Figure 10:**
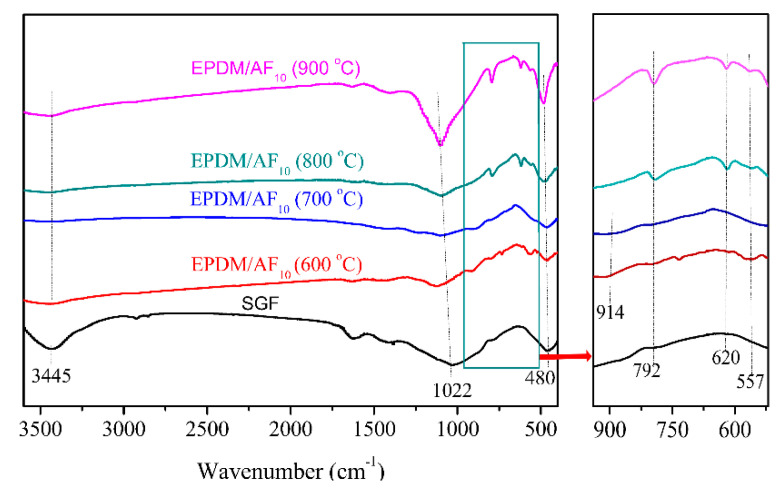
FTIR spectra of silicate glass frits (SGF) and residues of EPDM/AF_10_ fired at different temperatures.

To identify the phase composition of the residue, XRD and FTIR were performed for the residues formed at various temperatures, and the results are shown in Figure 9 and Figure 10. It is shown that an obvious “hump” appeared at 15°–35°, indicating the amorphous structure [45,46] of EPDM/AF_10_ after firing at 600 °C. Meanwhile, there are some weak peaks appearing at the 2θ of 20.09° and 21.83° represented sodium phosphate (Pdf No.11–383) and cristobalite (Pdf No.1–438), respectively [23,47]. With increasing temperature, the “hump” became very weak, and even disappeared. When EPDM/AF_10_ was fired at 700 °C or even higher, the main crystalline phase of its residues was cristobalite. For the FTIR curve, the peak at 1022 cm^−1^ is assigned to the vibration of Si-O-Si in SGF [48,49,50]. The absorbing peak at 3445 cm^−1^ is ascribed to the stretching vibration of the O-H bond [51], which gradually decreased when the temperature increased. These changes meant a change in the SGF structure. For ceramic residues formed at 600 °C and 700 °C, it was found that some new peaks appeared at 914 cm^−1^ and 557 cm^−1^, which was ascribed to the formation of the sodium phosphate [52]. Moreover, with the temperature increasing, a new peak appeared at 620 cm^−1^, and the peak at 792 cm^−1^ became stronger in the FTIR spectrum of residues of EPDM/AF_10_, representing the formation of cristobalite [53]. These results were consistent with the analysis of XRD, and illustrated that the phase change occurred when the EPDM/AF_10_ composite was fired at higher temperatures. Thus, the composite showed a good self-supporting property even if the firing temperature was higher than the melting point of the SGF.

**Figure 11:**
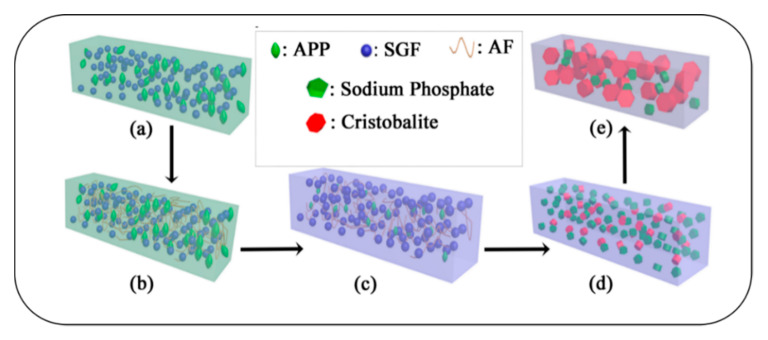
Schematic presentation of the mechanism of self-supporting: (**a**) ceramifiable EPDM composite without AF, (**b**) EPDM/AF composite, (**c**) fired at below 600 °C, (**d**) fired at a range of 600–800 °C, and (**e**) fired at above 800 °C.

The formation mechanism of this novel ceramifiable EPDM composite was proposed as shown in Figure 11. Below about 600 °C, the ceramifiable EPDM composite can support its own weight very well with the AF network structures. The formation of crystalline phases (sodium phosphate and cristobalite) played a significant role in keeping the self-supporting property of the ceramifiable EPDM composite above 600 °C.

## 4. Conclusions

A ceramifiable EPDM composite with fiber network structures was successfully prepared. AF as a “skeletal structure” existed in the ceramifiable EPDM composite system. The AF network structure reinforced the interaction between EMPD and fillers. Compared with the ceramifiable EPDM composite without AF, the tear strength of EPDM/AF_15_ increased from 11.53 to 22.02 N/m. The solvent resistance and ablative resistance of the ceramifiable EPDM composite with AF were also enhanced. With the addition of AF, the ceramifiable and self-supporting properties of the ceramifiable EPDM composite were improved significantly. Compared with the residue of ceramifiable EPDM composite without AF, the residue of EPDM/AF system had higher flexural strength and a more compact structure. The excellent self-supporting property of the EPDM/AF system was obtained by combining the formation of the AF network and the formation of crystalline phases at higher temperature (above 600 °C). This study provides a new thought and route to prepare ceramifiable polymer composites with excellent overall performance, which will be of great theoretical and practical significance.

## Figures and Tables

**Table 1:** Formulations of ceramifiable EPDM composites (g).

Sample	EPDM	SGF	APP	AF	DCP	S
EPDM/AF_0_	70	85	45	0	2.10	0.35
EPDM/AF_5_	70	85	45	3.5	2.10	0.35
EPDM/AF_10_	70	85	45	7	2.10	0.35
EPDM/AF_15_	70	85	45	10.5	2.10	0.35

**Table 2:** Curing characteristics of pure EPDM at different temperatures.

Sample	*t*_10_ (s)	*t*_90_ (s)	*M*_L/_(N·m)	*M*_H/_(N·m)	Δ*M*/(N·m)
EPDM-160 °C	127	689	0.28	0.57	0.29
EPDM-170 °C	102	651	0.12	0.59	0.47
EPDM-180 °C	70	388	0.11	0.65	0.53

**Table 3:** Curing characteristics of ceramifiable EPDM composites at 180 °C.

Sample	*t*_10_ (s)	*t*_90_ (s)	*M*_L/_(N·m)	*M*_H/_(N·m)	Δ*M*/(N·m)
EPDM/AF_0_	84	559	0.39	1.72	1.33
EPDM/AF_5_	86	586	0.56	2.07	1.51
EPDM/AF_10_	86	650	0.62	2.23	1.61
EPDM/AF_15_	87	638	0.62	2.48	1.86

**Table 4:** Liquid absorbency and dimensional change of ceramifiable EPDM composites in pure tetrachloromethane.

Sample		Dimensional Change (Length × Width) (mm)	Liquid Absorbency (%)
**EPDM/AF_0_**	Before swelling	20.08 × 20.06	252.23
	After swelling	29.34 × 29.34	
EPDM/AF_5_	Before swelling	20.08 × 20.18	184.25
	After swelling	21.24 × 24.50	
EPDM/AF_10_	Before swelling	20.04 × 20.14	150.72
	After swelling	21.10 × 23.20	
EPDM/AF_15_	Before swelling	20.02 × 20.04	132.66
	After swelling	20.90 × 20.90	

**Table 5:** Mechanical properties of ceramifiable EPDM composites.

Sample	Tearing Strength (N/m)
EPDM/AF_0_	11.53 ± 0.62
EPDM/AF_5_	14.85 ± 0.70
EPDM/AF_10_	16.61 ± 0.95
EPDM/AF_15_	22.02 ± 0.65

**Table 6:** Combustion test results of ceramifiable EPDM composites.

Sample	Dripping or Not	UL94 Rating
EPDM/AF_0_	Dripping	NC
EPDM/AF_5_	Dripping	NC
EPDM/AF_10_	No dripping	NC
EPDM/AF_15_	No dripping	NC

NC: not classified without self-extinguishing.

**Table 7:** Bending angles for the residues of ceramifiable EPDM composites firing at different temperatures.

Sample	Bending Angle at 600 °C (°)	Bending Angle at 700 °C (°)	Bending Angle at 800 °C (°)	Bending Angle at 900 °C (°)
EPDM/AF_0_	16.32	67.42	90	90
EPDM/AF_5_	10.63	12.46	21.65	39.91
EPDM/AF_10_	0	0	5.37	9.6
EPDM/AF_15_	0	0	0	6.38

**Table 8:** Linear ablation rate of ceramifiable EPDM composites.

Sample	Linear Ablation Rate (mm/s)
EPDM/AF_0_	- ^a^
EPDM/AF_5_	0.28
EPDM/AF_10_	0.25
EPDM/AF_15_	0.23

^a^ Sample was burned through.

**Table 9:** TGA data of AF, EPDM/AF_0_ and EPDM/AF_10_ under N_2_.

Sample	*T*_5%_ (°C)	*T*_max1_ (°C)	Residue at 500 °C (wt %)
AF	481.23	-	94.49
EPDM/AF0	360.73	381.57	56.74
EPDM/AF10	378.39	394.16	57.62

## Supplementary Materials

The following are available online at https://www.mdpi.com/2073-4360/12/7/1523/s1, Figure S1: Photos of surface morphology of samples EPDM/AF0 (left) and EPDM/AF10 (right) fired at 600 °C (a), 700 °C (b), 800 °C (c) and 900 °C (d); Figure S2: Digital photos of AF before and after heat reatment at 400 °C; Figure S3: Photographic image of the back side and front side of the composites after ablation rate test.
