# Supplementary material for: Improved Self-Supporting and Ceramifiable Properties of Ceramifiable EPDM Composites by Adding Aramid Fiber
Source: Polymers (Basel). 2020 Jul 9;12(7):1523. doi: 10.3390/polym12071523 (PMC7408063; doi:10.3390/polym12071523)
Supplement: Supplementary file 1 [file polymers-12-01523-s001.pdf]

Supporting Information

Improved self-supporting and ceramifiable properties of ceramifiable EPDM composites by adding aramid fiber

Dong Zhao [1,2], Wei Liu [1,2], Yucai Shen [1,3,*], Guodong Jiang [1,3] and Tingwei Wang [1,3,*]

[1] College of Material Science and Engineering, Nanjing Tech University, Nanjing 211816, China; 8113557343@njtech.edu.cn (D.Z.); 201861103043@njtech.edu.cn (W.L.); gdjiang@njtech.edu.cn (G.J.)

[2] Jiangsu Collaborative Innovation Center for Advanced Inorganic Function Composites, Nanjing 211816, China

[3] Suqian Advanced Materials Institute of Nanjing Tech University, Suqian 223800, China

* Correspondence: ycshen@njtech.edu.cn (Y.S.); wangtw@njtech.edu.cn (T.W.); Tel.: +86-159-5182-6321 (Y.S.); +86-136-0158-5533 (T.W.)

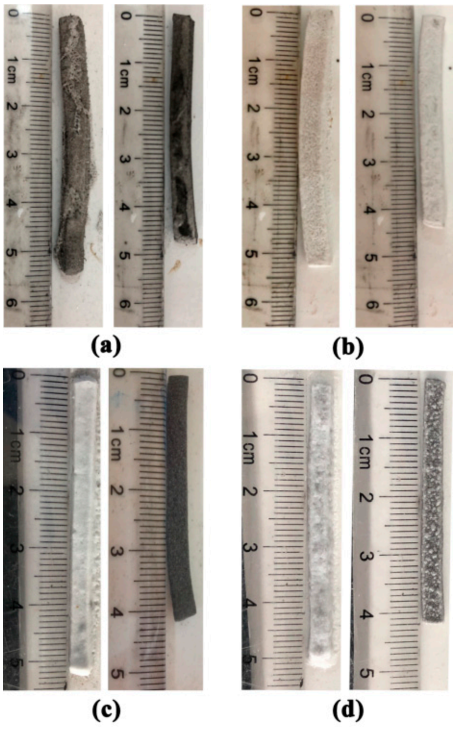

Figure S1. Photos of surface morphology of samples EPDM/AF_0 (left) and EPDM/AF_{10} (right) fired at 600 °C (a), 700 °C (b), 800 °C (c) and 900 °C (d).

Figure S1 shows that the surface of sample EPDM/AF_0 is rough, while sample EPDM/AF_{10} without obvious pores and cracks after firing at 600 or 700 °C. What more, compared with sample EPDM/AF_0, sample EPDM/AF_{10} has an obvious shrinkage after firing at 600, 700, 800 and 900 °C.

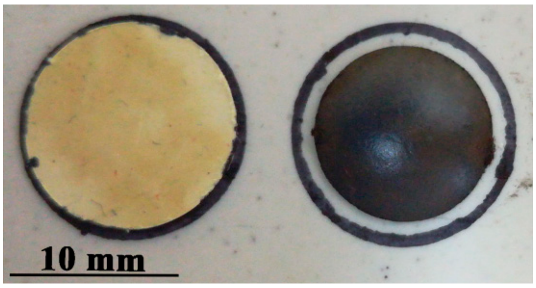

Figure S2. Digital photos of AF before and after heat reatment at 400 °C.

The AF was poured into molds, compressed at room temperature for 30 min under 20 MPa pressure. Figure S2 shows that, after the heat treating, the oriented AF sheet has an obvious shrinkage.

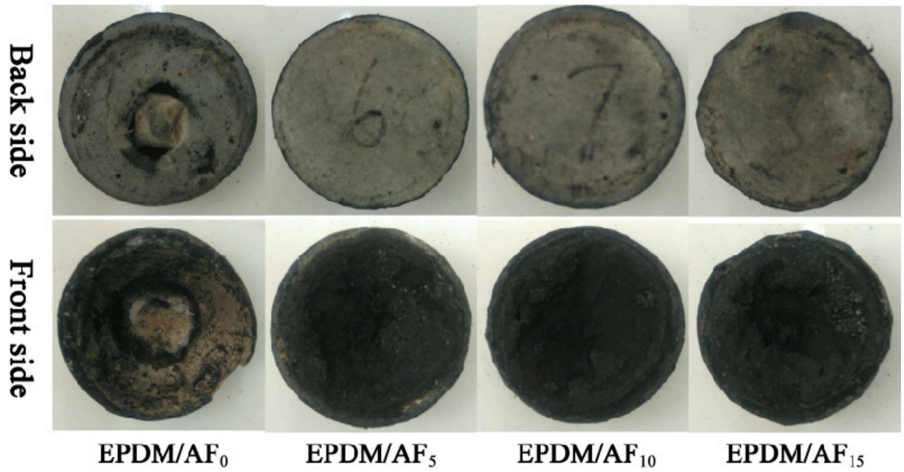

Figure S3. Photographic image of the back side and front side of the composites after ablation rate test.
